# Ferroptosis-associated DNA methylation signature predicts overall survival in patients with head and neck squamous cell carcinoma

**DOI:** 10.1186/s12864-022-08296-z

**Published:** 2022-01-18

**Authors:** Yuanyuan Xu, Min Hong, Deyu Kong, Jun Deng, Zhaoming Zhong, Jin Liang

**Affiliations:** 1 Department of Oncology, First People’s Hospital of Kunming, Kunming, 650000 China; 2 Department of Medical Oncology, First Affiliated Hospital of Kunming Medical University, Kunming, 650000 China

**Keywords:** Ferroptosis, DNA methylation signature, Head and neck squamous cell carcinoma, Oral squamous cell carcinoma, Diagnostic indicator

## Abstract

**Background:**

Head and neck squamous cell carcinoma (HNSCC) is a common cancer characterized by late diagnosis and poor prognosis. The aim of this study was to identify a novel ferroptosis-related DNA methylation signature as an alternative diagnosis index for patients with HNSCC.

**Methods:**

Methylome and transcriptome data of 499 HNSCC patients, including 275 oral squamous cell carcinoma (OSCC) samples, were obtained from The Cancer Genome Atlas (TCGA). An additional independent methylation dataset of 50 OSCC patients from the NCBI Gene Expression Omnibus (GEO) database was used for validation. As an index of ferroptosis activity, the ferroptosis score (FS) of each patient was inferred from the transcriptome data using single-sample gene set enrichment analysis. Univariate, multivariate, and LASSO Cox regression analyses were used to select CpG sites for the construction of a ferroptosis-related DNA methylation signature for diagnosis of patients.

**Results:**

We initially inferred the FS of each TCGA HNSCC patient and divided the samples into high- and low-FS subgroups. Results showed that the high-FS subgroup displayed poor overall survival. Moreover, 378 differentially methylated CpG sites (DMCs) were identified between the two HNSCC subgroups, with 16 selected to construct a 16-DNA methylation signature for risk prediction in HNSCC patients using the LASSO and multivariate Cox regression models. Relative operating characteristic (ROC) curve analysis showed great predictive efficiency for 1-, 3-, and 5-year HNSCC survival using the 16-DNA methylation signature. Its predictive efficiency was also observed in OSCC patients from the TCGA and GEO databases. In addition, we found that the signature was associated with the fractions of immune types in the tumor immune microenvironment (TIME), suggesting potential interactions between ferroptosis and TIME in HNSCC progression.

**Conclusions:**

We established a novel ferroptosis-related 16-DNA methylation signature that could be applied as an alternative tool to predict prognosis outcome in patients with HNSCC, including OSCC.

**Supplementary Information:**

The online version contains supplementary material available at 10.1186/s12864-022-08296-z.

## Background

Head and neck squamous cell carcinoma (HNSCC) is one of the most common malignancies worldwide and is associated with high morbidity and mortality [1]. According to the 2018 Global Cancer Report, there are 890,000 new HNSCC cases worldwide each year as well as 450,000 deaths [2]. HNSCC is a heterogeneous disease with marked clinical, phenotypical, pathological, and biological differences [3]. Most tumors develop in the mucous membranes of the oral cavity, pharynx, and larynx [1]. At present, the overall 5-year survival of HNSCC across all stages is ~ 60% [4, 5]. Notably, despite considerable effort and advances in multimodal therapy, over 60% of patients diagnosed with HNSCC present at a locally advanced stage with a 5-year survival rate of less than 30% [6]. Therefore, the identification of reliable prognostic biomarkers is urgently needed for early detection, effective treatment, and improved prognosis in HNSCC patients.

Programmed cell death (PCD) is a key biological process in animal development and tissue homeostasis [7]. Mounting evidence suggests that PCD plays vital roles in tumorigenesis, progression, and metastasis [8, 9]. Ferroptosis is a new form of PCD that differs from traditional cell death modes (e.g., apoptosis and necrosis) and is iron and reactive oxygen species (ROS) dependent [9–11]. Furthermore, it is an emerging concept in cancer biology and an attractive intervention target for precision cancer therapy [12]. Consistently, dynamic expression of ferroptosis-related genes (e.g., *GPX4* and *SLC7A11*) is reported in both tumor progression and suppression [13–15]. In addition, DNA methylation, a well-studied type of epigenetic modification, functions in the regulation of gene transcription [16]. Aberrations in DNA methylation play an important role in pathogenesis of cancer [17]. For instance, transcriptional silencing of tumor suppressor genes by promoter hypermethylation has been noted in most, if not all, cancers, including HNSCC [18, 19]. Currently, DNA methylation is widely used as a diagnostic, predictive, and prognostic biomarker for multiple cancers (e.g., breast and lung cancer) due to its advanced stability, frequency, and accessibility characteristics [20]. Moreover, emerging evidence provides support for the role of DNA methylation in controlling the expression of ferroptosis-related genes (e.g., *GPX4*) [21], highlighting the potential of ferroptosis-related DNA methylation signatures as promising biomarkers in HNSCC risk stratification.

In this study, we collected methylome, transcriptome, and clinical data of HNSCC patients from The Cancer Genome Atlas (TCGA). Furthermore, as a measure of ferroptosis activity, we also determined the ferroptosis score (FS) for each HNSCC patient based on the expression of ferroptosis-related genes using single-sample gene set enrichment analysis (ssGSEA). The HNSCC patients were divided into two subgroups (i.e., high- and low-FS). The high-FS HNSCC subgroup displayed poor survival probability. We next identified differentially methylated CpG sites (DMCs) between the high-FS and low-FS HNSCC subgroups. A 16-DNA methylation signature was constructed for the diagnosis of HNSCC patients using LASSO and multivariate Cox regression analyses. Kaplan-Meier survival and receiver operating characteristic (ROC) curve analyses of independent datasets validated that the 16-DNA methylation signature could be an effective diagnosis biomarker for HNSCC, including OSCC. In addition, we found that the ferroptosis-related signature had a bearing on the tumor immune microenvironment (TIME) of HNSCC patients.

## Methods

### Data collection and preprocessing

Transcriptome (RNA-seq, HTSeq-Counts/FPKMs) and methylome (Illumina Human Methylation 450, HM450) data of 499 HNSCC patients and 44 normal controls, as well as related clinical information, were download from the TCGA database by UCSC Xena (https://xena.ucsc.edu/). The human papillomavirus (HPV) infection status of 179 HNSCC patients was obtained from the cBioPortal for Cancer Genomics (https://www.cbioportal.org/). The gene expression level (FPKM) and DNA methylation level (*β*-value) were normalized using the ‘*normalize.quantiles*’ function in the R “preprocessCore” package. The TCGA HNSCC-OSCC patients were chosen based on the terms ‘Base of tongue’, ‘Floor of mouth’, ‘Gum’, ‘Other and unspecified parts of mouth’, ‘Other and unspecified parts of tongue’, and ‘Palate’ in the clinical information. An additional independent HM450 methylation dataset of 50 OSCC patients was downloaded from the NCBI Gene Expression Omnibus (GEO) database (access number: GSE52793). The fractions of 22 types of infiltrating immune cells in HNSCC patients were collected from the Cancer Immunome Atlas (TICA, https://tcia.at/), based on the CIBERSORT algorithm [22].

### Survival analysis

Univariate and multivariate Cox proportional hazards analyses were performed using the “survival” package. Survival curves were drawn using the “survminer” R package with the Kaplan-Meier (KM) estimator.

### Inference of FS

Ferroptosis-related genes were collected from the FerrDb database, and included 108 drivers, 69 suppressors, and 111 markers [23]. Univariate Cox survival analysis identified genes showing positive and negative associations between gene expression and overall survival in HNSCC, defined as ferroptosis-positive and -negative components, respectively. The method to infer sample FS followed that of Liu et al. [24]. Enrichment score on the gene set with positive and negative associations between their expression and HNSCC survival was calculated using ssGSEA in the “GSVA” package, respectively [25], and the difference between negative and positive components was defined as the FS of a patient. A high FS represented low ferroptosis activity.

### DMC analysis

The DMCs between the two groups were identified using the “limma” package by modeling study variables (i.e., case-control status) [26]. An adjusted *P-*value < 0.01 was considered statistically significant.

### Selection of ferroptosis-related methylation sites

LASSO Cox regression analysis was performed using the “glmnet” package to select ferroptosis-related methylation sites relevant to HNSCC survival. After that, the methylation sites were used as covariates to construct the multivariate Cox proportional hazards model. The ROC curve was drawn using the “plotROC” package and the area under the ROC curve (AUC) was calculated using the “survivalROC” package. Statistical analyses were conducted using the R platform (v4.0.3).

## Results

### Inferring FSs of HNSCC patients based on transcriptome data

**Fig. 1:**
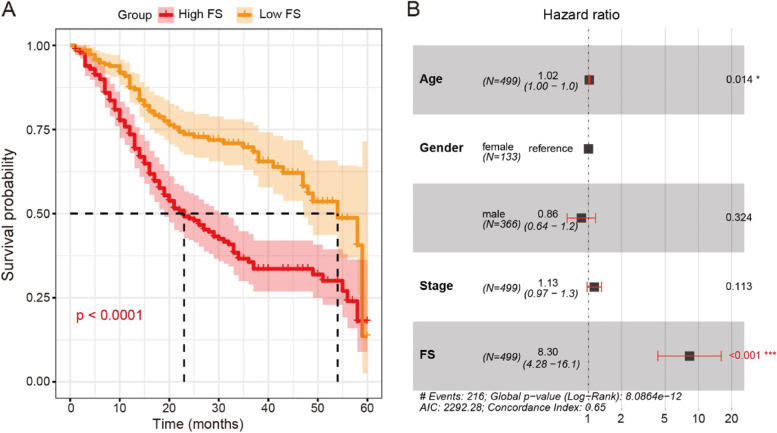
Associations between FS and HNSCC overall survival. **A** Kaplan-Meier survival curve of HNSCC with high and low FS. **B** Forest plot of association between FS and HNSCC survival using multivariate Cox proportional hazards model

To assess ferroptosis activity in HNSCC, we collected ferroptosis-related genes from the FerrDb database [23]. Using the univariate Cox model, we identified 13 and 34 genes showing positive and negative associations, respectively, between gene expression and overall survival in HNSCC (*P* < 0.05; Supplementary Table 1). We inferred the FSs of HNSCC patients as an index of ferroptosis activity based on the expression levels of the above two gene sets using ssGSEA (see Methods). The FSs ranged from − 0.62 to 0.84. Here, we divided the HNSCC patients into two subgroups based on median FS and performed Kaplan-Meier survival analysis. Results showed that the high-FS HNSCC patients displayed poor overall survival (Fig. 1A). We further conducted multivariate Cox model analysis and confirmed the association between FSs and HNSCC survival after correcting the effects of covariates, i.e., age, sex, and pathological stage (Fig. 1B).

### Ferroptosis-related DNA methylation signature associated with HNSCC survival

We initially identified 20,381 DMCs between HNSCC patients and normal samples based on *P* < 0.01 and methylation difference > 0.2. Likewise, 1248 DMCs were identified between high- and low-FS HNSCC patients based on an adjusted *P* < 0.01 and methylation difference > 0.1. We identified a total of 378 CpG sites showing methylation differences between HNSCC patients and normal samples, and between high- and low-FS HNSCC subgroups.

**Fig. 2:**
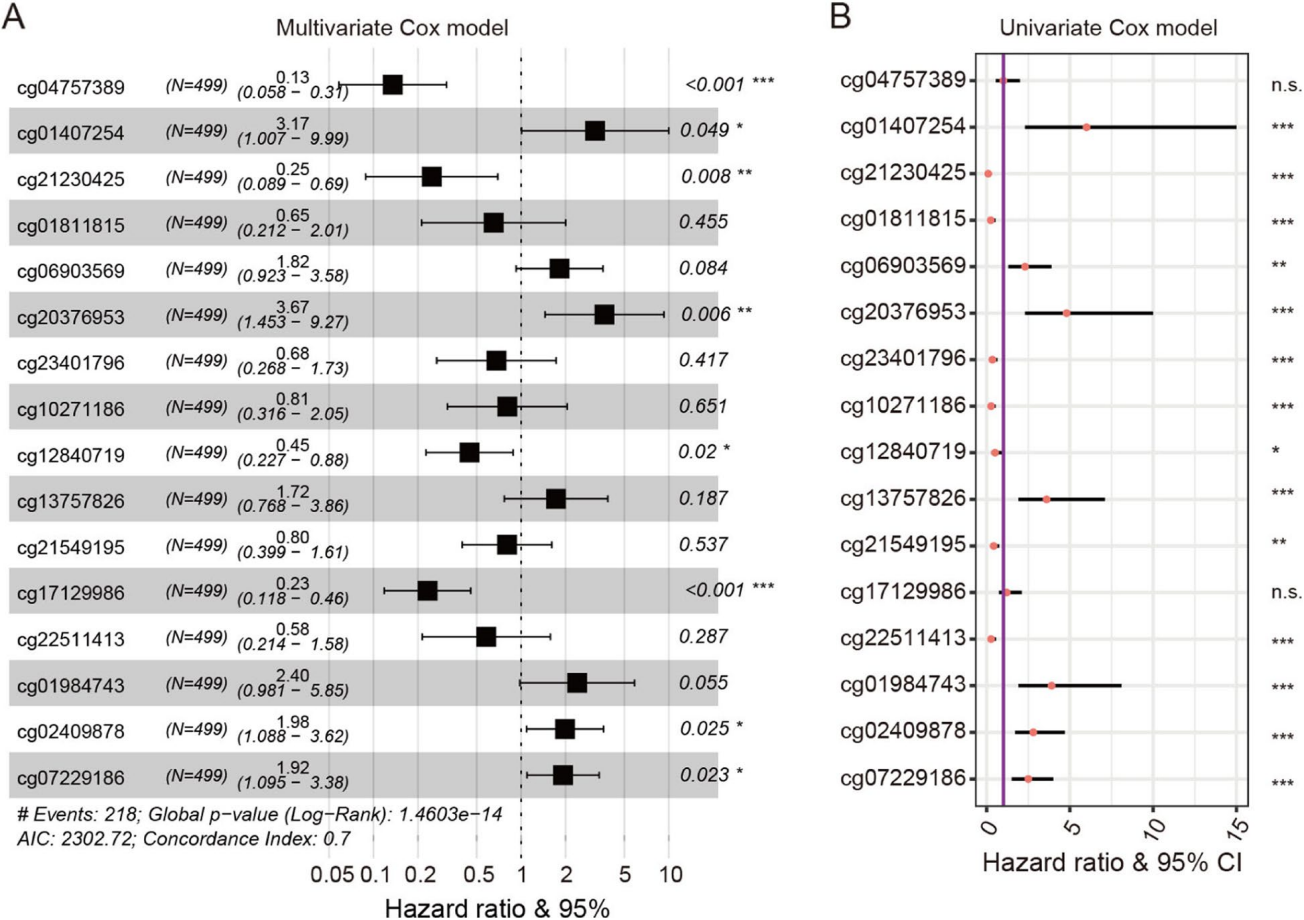
Cox proportional hazards regression analysis for CpG sites of 16-DNA methylation signature in HNSCC patients. **A** Association between methylation level of each site and HNSCC survival by multivariate Cox model. **B** Association between methylation level of each site and HNSCC survival by univariate Cox model (*: *P* < 0.05; **: *P* < 0.01; ***: *P* < 0.001; n.s.: non-significant)

To identify a DNA methylation signature for the prediction of overall survival in HNSCC, we input the 378 DMCs in the LASSO Cox regression model to narrow the DMC range (see Methods). A total of 16 CpG sites with coefficient values not equal to 0 were selected. We then recalculated the coefficient values of these 16 CpG sites using the multivariate Cox model (Fig. 2A). Based on the methylation level and coefficient value of each CpG site, a risk score model was constructed as follows: risk score = (− 2.0025*cg04757389) + (1.1545*cg01407254) + (− 1.3936*cg21230425) + (− 0.4286*cg01811815) + (0.5984*cg06903569) + (1.3003*cg20376953) + (− 0.3857*cg23401796) + (− 0.2162*cg10271186) + (− 0.8047*cg12840719) + (0.5441*cg13757826) + (− 0.2200*cg21549195) + (− 1.4603*cg17129986) + (− 0.5432*cg22511413) + (0.8739*cg01984743) + (0.6851*cg02409878) + (0.6541*cg07229186). In addition, univariate Cox regression was performed to assess the correlation between each CpG site and HNSCC survival (Fig. 2B and Supplementary Table 2). Based on the multivariate and univariate Cox models, the 16 CpG sites showed a significant association between their methylation and HNSCC survival. Results showed that the risk scores estimated by the signature were significantly correlated with FSs in the HNSCC patients (*P* = 2.46e-31; Supplementary Fig. 1).

We also investigated the potential roles of the 16 CpG sites of the DNA methylation signature in regulating the expression of 45 ferroptosis-related genes associated with HNSCC survival. DNA methylation-gene expression correlation analyses were conducted. Results showed that each of the 16 DMCs were correlated with a number of ferroptosis-related genes, ranging from 2 to 16 (Supplementary Fig. 2). This finding indicates that the 16 CpG sites likely modulate transcription of ferroptosis-related genes either directly or indirectly.

### Evaluation of risk model for prediction of HNSCC survival

**Fig. 3:**
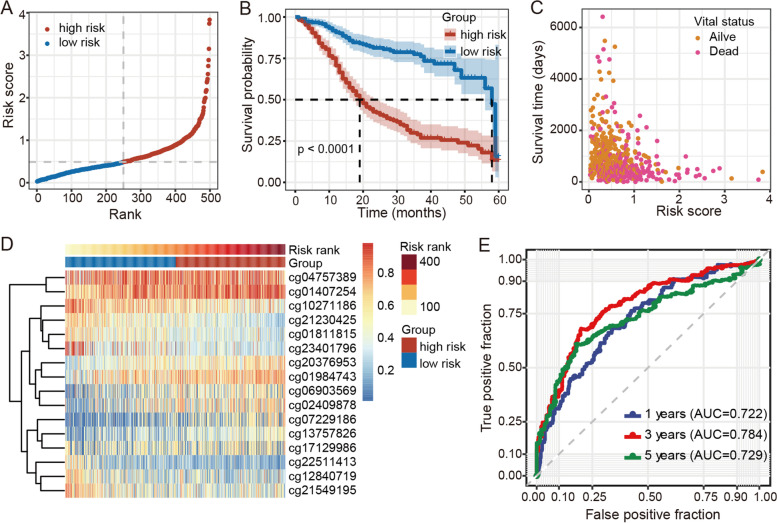
Construction of ferroptosis-related DNA methylation signature using methylation of 16 CpG sites in HNSCC patients. **A** 16-DNA methylation signature risk score distribution. **B** Survival curves for HNSCC patients with high- and low-risk scores. **C** Vital status of HNSCC patients in high- and low-risk groups. **D** Heatmap of methylation level of 16-DNA methylation signature in HNSCC patients with high- and low-risk scores. **E** ROC curves for 1-, 3-, and 5-year overall survival in HNSCC patients

To evaluate the survival assessment model, we calculated the risk score of each HNSCC patient, then divided the HNSCC patients into high- and low-risk groups based on their median risk score (Fig. 3A). We then conducted Kaplan-Meier survival analysis and found that HNSCC patients with high-risk scores had poorer survival probability (Fig. 3B), suggesting that the risk score model may be an effective prognostic predictor. As shown in Fig. 3C, there were more death cases in the high-risk group than in the low-risk group. Additionally, the heatmap showed the methylation level of the 16 CpG sites in the prognostic model (Fig. 3D). The ROC curves for HNSCC patients at 1, 3, and 5 years are shown in Fig. 3E. The AUCs were 0.722 at 1 year, 0.784 at 3 years, and 0.729 at 5 years.

### Evaluation of 16-DNA methylation signature for OSCC survival

**Fig. 4:**
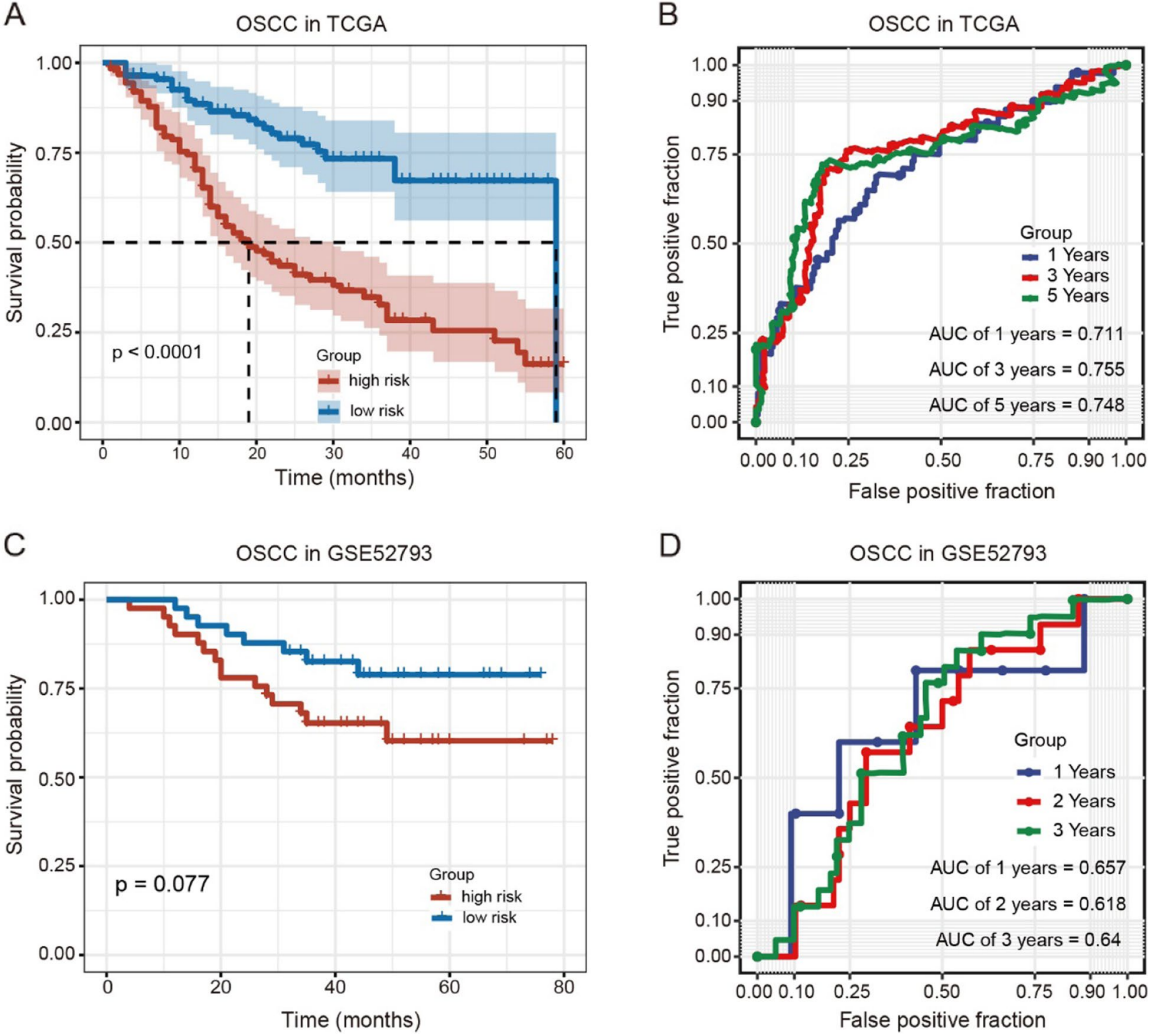
16-DNA methylation signature in OSCC patients. **A** Survival curves for OSCC patients from TCGA with high- and low-risk scores. **B** ROC curves for 1-, 3-, and 5-year overall survival in OSCC patients from TCGA. **C** Survival curves for OSCC patients from GSE52793 with high- and low-risk scores. **D** ROC curves for 1-, 2-, and 3-year overall survival in OSCC patients from GSE52793

As OSCC is representative of most HNSCC [27], we next investigated the OSCC cases in HNSCC patients. Consistently, OSCC patients with high-risk scores displayed poorer survival probability (Fig. 4A), with AUCs of 0.711 at 1 year, 0.755 at 3 years, and 0.748 at 5 years (Fig. 4B). To further examine the prognostic value of the 16-DNA methylation signature in another cohort, we collected an independent DNA methylation dataset of OSCC patients. As expected, OSCC patients with high-risk scores presented poorer survival probability, with AUCs of 0.657 at 1 year, 0.618 at 2 years, and 0.640 at 3 years (Fig. 4C and D).

### Comparison of TIME between high- and low-risk HNSCC patients

**Fig. 5:**
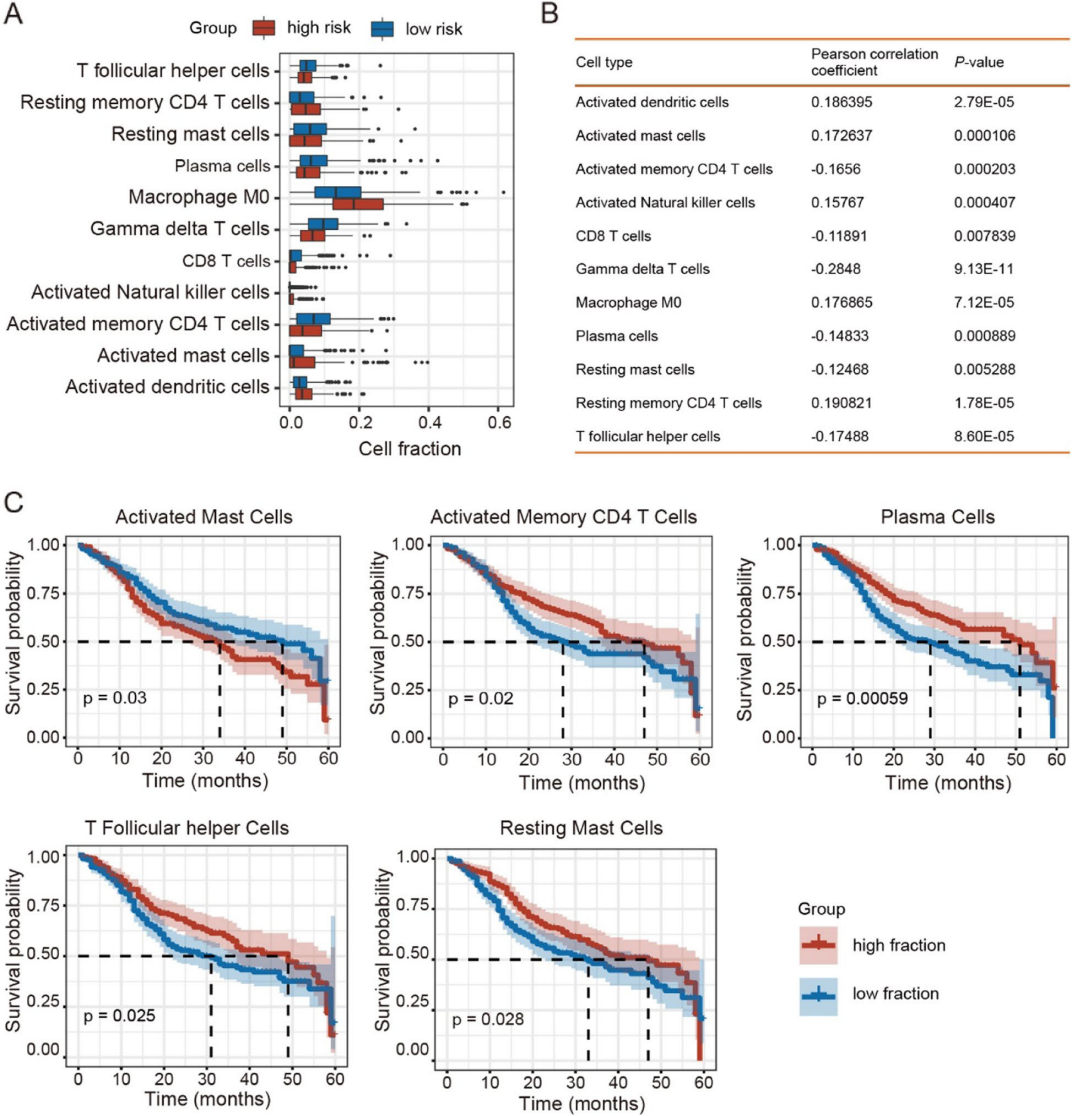
Association between fraction of immune cells and FSs. **A** Fraction differences in immune cells between high-risk and low risk HNSCC subgroups. **B** Correlation between fractions of immune cells and FSs. **C** Survival curves for HNSCC patients with high fraction and low fraction of various immune cell types

Given the crosstalk between ferroptosis and TIME [28, 29], we next determined whether the ferroptosis-associated 16-DNA methylation signature was involved in tumor-infiltrating immune cells in the TIME of HNSCC patients. A total of 22 immune cell types from HNSCC patients were available from the TICA (https://tica.at). Results showed that the fractions of resting memory CD4 T cells, macrophages M0, activated natural killer cells, activated mast cells, and activated dendritic cells increased in HNSCC with high-risk scores, whereas the fractions of T follicular helper cells, resting mast cells, plasma cells, gamma delta T cells, CD8 T cells, and activated memory CD4 T cells decreased in HNSCC with low-risk scores (Fig. 5A). Moreover, all 11 immune cell types showed a significant association between their fraction and FS (Fig. 5B). Accordingly, further survival analyses showed that the fraction of activated mast cells was associated with poor HNSCC survival, while the fractions of activated memory CD4 T cells, plasma cells, T follicular helper cells, and resting mast cells were associated with good HNSCC survival (*P* < 0.05; Fig. 5C). These findings indicate the 16-DNA methylation signature involving ferroptosis may play an important role in HNSCC progression and classification via TIME regulation.

## Discussion

Ferroptosis is an iron-dependent form of non-apoptotic-regulated cell death that shows promise in tumor treatment [30]. Ferroptosis activity evaluated by ferroptosis gene expression is effective in tumor diagnosis and therapy, including in HNSCC [31, 32]. DNA methylation, a well-studied form of epigenetic modification, regulates ferroptosis by modulating the transcription of corresponding genes (e.g., *GPX4*) in tumors [21]. In addition, DNA methylation is widely used as a biomarker in healthcare applications due to its relative stability and accuracy [33]. Therefore, a ferroptosis-related DNA methylation signature could be an effective alternative tool for prognosis and diagnosis of HNSCC patients.

Here, we analyzed methylome and transcriptome data of HNSCC patients from the TCGA. A novel FS for each HNSCC patient was initially inferred based on the expression of 47 genes associated with ferroptosis and HNSCC survival. Among them, several genes are linked to ferroptosis and tumorigenesis. For instance, *ATG5* is considered a contributor for inducing ferroptosis [34], and its high expression promotes tumor metastasis [35]; *BAP1* encodes a nuclear deubiquitinating enzyme, which plays an important role in ferroptosis and tumor suppression [36]. Further survival analysis showed that high-FS HNSCC patients exhibited poor survival probability (*P* < 0.05), suggesting a high correlation between FS and HNSCC survival.

We next identified 378 DMCs between the high- and low-FS subgroups of HNSCC patients, and further selected 16 DMCs using the LASSO Cox regression model to construct a DNA methylation signature for diagnosis and prediction of HNSCC. Among the 16 DMCs, 9 were also selected by using a robust network-based variable selection approach, a novel algorithm addressed the skewed distribution and outlier in survival time data [37, 38], indicating the robustness of the methylation signature. ROC curve analysis revealed that this signature had great predictive efficiency for HNSCC survival. Further analyses revealed that the 16-DNA methylation signature well predicted OSCC patient survival in the two cohorts. Moreover, the 16 DMCs displayed significant associations between their methylation level and expression of ferroptosis-related genes, thus suggesting potential roles in the regulation of ferroptosis activity in tumors. In addition, ROC curve and heatmap showed that the 16-DNA methylation signature also performed well in the classification of HPV-positive and HPV-negative HNSCC subgroups, and the AUC value was equal to 0.87 (Supplementary Fig. 3A and B), suggesting that the methylation signature has potential in predicting HPV status of HNSCC patients.

The 16-DNA methylation signature was also closely linked to the fractions of several immune cell types (i.e., activated mast cells, activated memory CD4 T cells, plasm cells, resting mast cells, and T follicular helper cells) that were associated with HNSCC survival. Among them, the high fraction of activated mast cells, which play pro-tumorigenic roles in several tumors [39], was associated with poor HNSCC survival. In contrast, high fractions of activated memory CD4 T cells and T follicular helper cells, which are known to have anti-tumor functions [40, 41], were associated with good HNSCC survival. These findings indicate that the ferroptosis-associated 16-DNA methylation signature may function in modulating HNSCC TIME.

## Conclusions

In summary, we constructed a ferroptosis-related 16-DNA methylation signature that could serve as an alternative biomarker to evaluate prognosis in HNSCC patients (including OSCC). In addition, the 16 CpG sites may serve as potential intervention targets for the selective induction of tumor cell death. However, further clinical and functional studies are required before the assay can be used for clinical purposes.

## Supplementary Information

**Additional file 1: Supplementary Figure 1.** The association between risk score and FS in HNSCC patients. **Supplementary Figure 2.** Correlation between methylation level of each site from 16-DNAm signature and expression of ferroptosis-related genes involving HNSCC survival. **Supplementary Figure 3.** Performance of the 16-DNA methylation signature in classifying HPV-positive and HPV-negative HNSCC patients. **Supplementary Table 1.** The ferroptosis-related genes associated with HNSCC survival. **Supplementary Table 2.** Univariate cox survival analysis for each site from the 16-DNA methylation signature in HNSCC patients.

## Data Availability

All data analysed during this study are included in this published article.

## Abbreviations

HNSCC: Head and neck squamous cell carcinoma
OSCC: Oral squamous cell carcinoma
TCGA: The Cancer Genome Atlas
PCD: Programmed cell death
ROS: Reactive oxygen species
FS: Ferroptosis score
ssGSEA: Single-sample gene set enrichment analysis
DMC: Differentially methylated CpG site
ROC: Receiver operating characteristic
TIME: Tumor immune microenvironment
AUC: Area under the ROC curve
