# Supplementary material for: Ferroptosis-associated DNA methylation signature predicts overall survival in patients with head and neck squamous cell carcinoma
Source: BMC Genomics. 2022 Jan 18;23:63. doi: 10.1186/s12864-022-08296-z (PMC8767683; doi:10.1186/s12864-022-08296-z)
Supplement: Supplementary file 1 — Additional file 1: Supplementary Figure 1. The association between risk score and FS in HNSCC patients. Supplementary Figure 2. Correlation between methylation level of each site from 16-DNAm signature and expression of ferroptosis-related genes involving HNSCC survival. Supplementary Figure 3. Performance of the 16-DNA methylation signature in classifying HPV-positive and HPV-negative HNSCC patients. Supplementary Table 1. The ferroptosis-related genes associated with HNSCC survival. Supplementary Table 2. Univariate cox survival analysis for each site from the 16-DNA methylation signature in HNSCC patients. [file 12864_2022_8296_MOESM1_ESM.docx]

**Supplementary Materials**


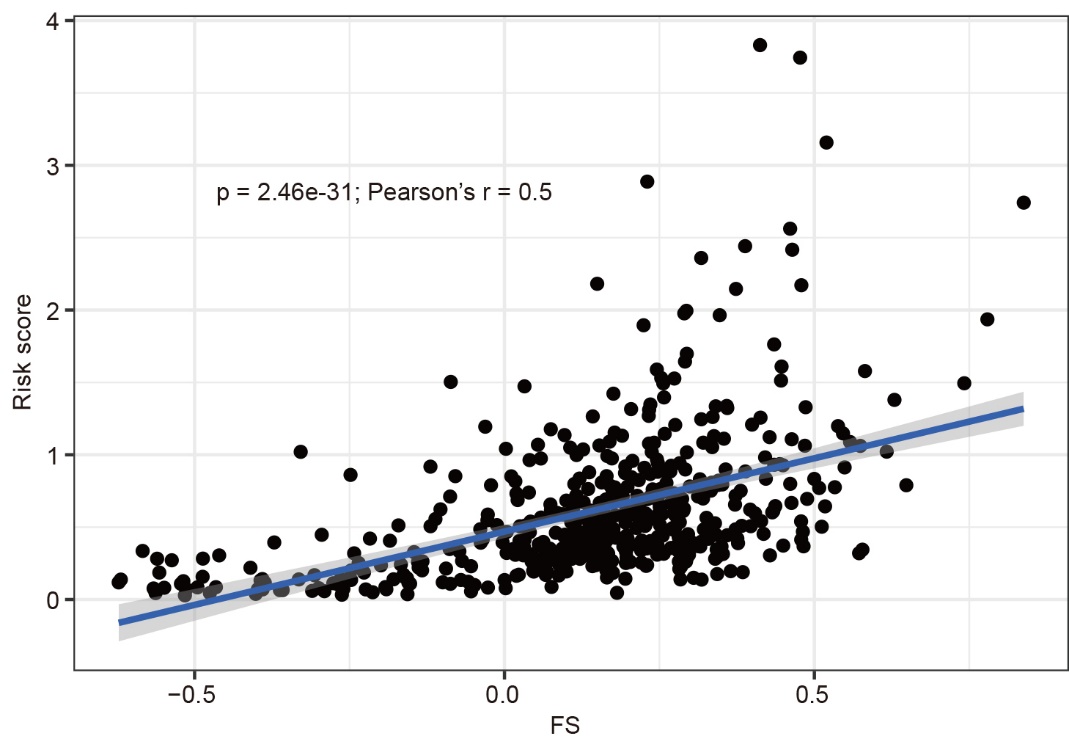


**Supplementary Figure 1. The association between risk score and FS in HNSCC patients.**


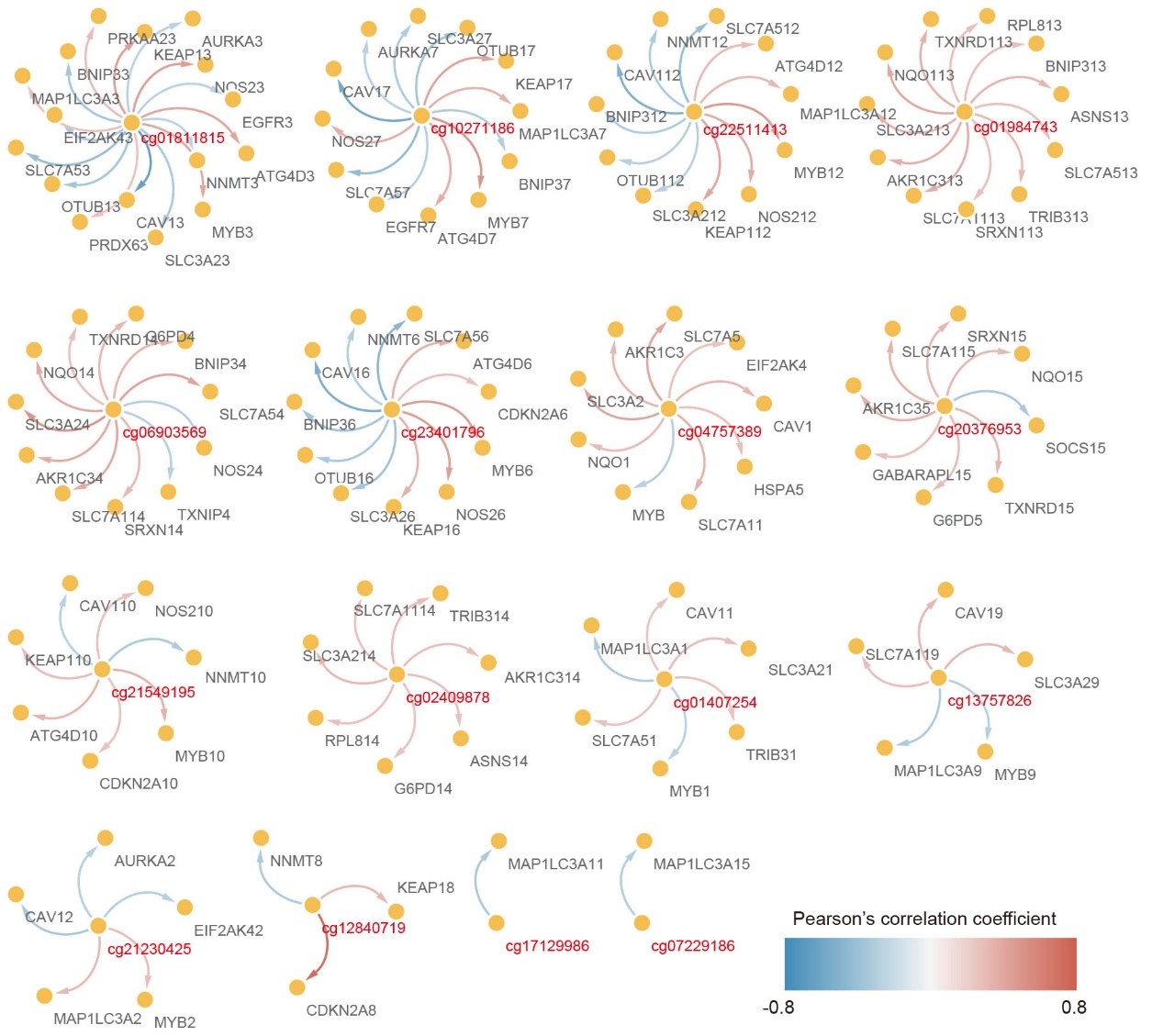


**Supplementary Figure 2. Correlation between methylation level of each site from 16-DNAm signature and expression of ferroptosis-related genes involving HNSCC survival.**


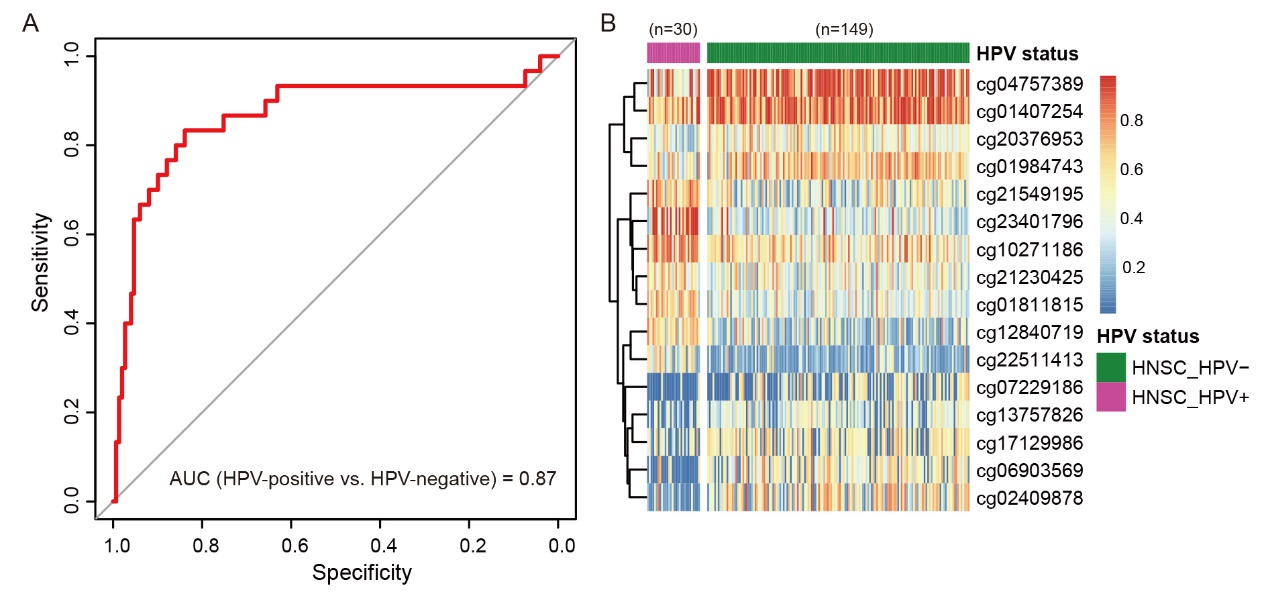


**Supplementary Figure 3. Performance of the 16-DNA methylation signature in classifying HPV-positive and HPV-negative HNSCC patients.** (A) ROC curve of the methylation signature in the classification of HPV-positive and HPV-negative HNSCC patients. (B) Heatmap for the methylation difference of the selected CpG sites between HPV-positive and HPV-negative patients.

**Supplementary Table 1. The ferroptosis-related genes associated with HNSCC survival.**

| Genes | HR (95% CI for HR) | *P*-value |
| --- | --- | --- |
| *Negative components* | | |
| ENSG00000151012.12 | 1.1 (1-1.2) | 0.041 |
| ENSG00000196139.10 | 1.1 (1-1.1) | 0.031 |
| ENSG00000181019.11 | 1.2 (1.1-1.3) | 0.00054 |
| ENSG00000167996.14 | 1.3 (1.1-1.5) | 0.0027 |
| ENSG00000168003.15 | 1.2 (1-1.4) | 0.033 |
| ENSG00000044574.7 | 1.4 (1.1-1.7) | 0.01 |
| ENSG00000165637.12 | 1.4 (1-1.9) | 0.034 |
| ENSG00000123983.12 | 1.3 (1-1.8) | 0.047 |
| ENSG00000167770.10 | 1.4 (1-1.9) | 0.032 |
| ENSG00000117592.8 | 1.4 (1.1-1.8) | 0.0089 |
| ENSG00000042286.13 | 1.3 (1.1-1.6) | 0.015 |
| ENSG00000105974.10 | 1.1 (1-1.2) | 0.032 |
| ENSG00000161016.14 | 1.2 (1-1.5) | 0.045 |
| ENSG00000160211.14 | 1.1 (1-1.3) | 0.033 |
| ENSG00000057663.11 | 1.8 (1.2-2.5) | 0.0019 |
| ENSG00000126581.11 | 1.5 (1-2.2) | 0.026 |
| ENSG00000034713.6 | 1.5 (1.1-2.1) | 0.019 |
| ENSG00000139112.9 | 1.2 (1-1.4) | 0.025 |
| ENSG00000146648.14 | 1.1 (1-1.2) | 0.044 |
| ENSG00000050748.16 | 1.9 (1.3-2.8) | 0.0024 |
| ENSG00000162409.9 | 1.3 (1.1-1.6) | 0.018 |
| ENSG00000163631.15 | 1.3 (1.1-1.6) | 0.035 |
| ENSG00000198431.14 | 1.1 (1-1.3) | 0.0087 |
| ENSG00000271303.1 | 1.2 (1-1.4) | 0.032 |
| ENSG00000176171.10 | 1.2 (1-1.4) | 0.012 |
| ENSG00000070669.15 | 1.3 (1.1-1.5) | 0.0016 |
| ENSG00000103257.7 | 1.1 (1-1.3) | 0.021 |
| ENSG00000101255.9 | 1.4 (1.2-1.6) | 0.00022 |
| ENSG00000074935.12 | 1.7 (1.1-2.8) | 0.033 |
| ENSG00000136244.10 | 1.1 (1-1.2) | 0.011 |
| ENSG00000059804.14 | 1.3 (1.1-1.5) | 0.00029 |
| ENSG00000128829.10 | 1.4 (1-2) | 0.046 |
| ENSG00000166741.6 | 1.1 (1-1.2) | 0.03 |
| ENSG00000087586.16 | 1.3 (1-1.6) | 0.019 |
| *Positive components* | | |
| ENSG00000168610.13 | 0.79 (0.63-0.99) | 0.041 |
| ENSG00000079999.12 | 0.7 (0.53-0.92) | 0.01 |
| ENSG00000130734.8 | 0.77 (0.6-0.98) | 0.035 |
| ENSG00000101460.11 | 0.85 (0.73-0.99) | 0.029 |
| ENSG00000147889.15 | 0.91 (0.84-0.97) | 0.0046 |
| ENSG00000185338.4 | 0.84 (0.72-0.97) | 0.019 |
| ENSG00000118513.17 | 0.79 (0.64-0.99) | 0.03 |
| ENSG00000163930.8 | 0.58 (0.41-0.83) | 0.003 |
| ENSG00000007171.15 | 0.82 (0.69-0.98) | 0.014 |
| ENSG00000265972.4 | 0.89 (0.81-0.99) | 0.031 |
| ENSG00000139718.9 | 0.69 (0.51-0.95) | 0.024 |
| ENSG00000197442.9 | 0.72 (0.58-0.9) | 0.0032 |
| ENSG00000137275.12 | 0.69 (0.5-0.96) | 0.032 |

**Supplementary Table 2. Univariate cox survival analysis for each site from the 16-DNA methylation signature in HNSCC patients.**

| CpG sites | HR (95% CI for HR) | P-value |
| --- | --- | --- |
| cg21230425 | 0.094 (0.042-0.21) | 4.00E-09 |
| cg20376953 | 4.8 (2.3-10) | 4.60E-05 |
| cg02409878 | 2.8 (1.7-4.7) | 5.40E-05 |
| cg01407254 | 6 (2.3-15) | 9.60E-05 |
| cg01984743 | 3.9 (1.9-8.1) | 9.80E-05 |
| cg13757826 | 3.6 (1.9-7.1) | 0.00011 |
| cg07229186 | 2.5 (1.5-4) | 0.00022 |
| cg10271186 | 0.26 (0.13-0.54) | 0.00025 |
| cg22511413 | 0.26 (0.12-0.56) | 3.00E-04 |
| cg01811815 | 0.24 (0.11-0.53) | 0.00031 |
| cg23401796 | 0.34 (0.18-0.65) | 0.00055 |
| cg06903569 | 2.3 (1.3-3.9) | 0.0025 |
| cg21549195 | 0.42 (0.24-0.76) | 0.0041 |
| cg12840719 | 0.5 (0.28-0.9) | 0.019 |
| cg17129986 | 1.2 (0.73-2.1) | 0.42 |
| cg04757389 | 1 (0.54-2) | 0.88 |
